# Analysis of the annual pollen integral in Albuquerque, New Mexico, shows a negative trend with temperatures for Juniper, Cottonwood, Elm, and Mulberry

**DOI:** 10.1007/s10453-022-09756-5

**Published:** 2022-09-26

**Authors:** Claudia M. Aprea, David J. Torres, Melany M. Cordova

**Affiliations:** Mathematics and Physical Science Department, Northern New Mexico College (NNMC), 921 N. Paseo de Oñate, Española, NM 87532, USA

**Keywords:** Allergies, Pollen, Juniper (*Juniperus, Cupressaceae*), Elm (*Ulmus*), Mulberry (*Morus*), Cottonwood (*Populus*), New Mexico

## Abstract

The goal of this study is to determine if the annual pollen integral (APIn) for the top tree allergens in the City of Albuquerque is correlated with meteorological variables. This analysis would be the first of its kind for this area. We used 17 consecutive years from 2004 to 2020 and data collected by the city of Albuquerque using a Spore Trap (Burkard) volumetric air sampler in a location designed to represent a typical desert environment. The pollen studied include Juniper, Elm, Ash, Cottonwood, and Mulberry. We found a negative linear correlation with early summer temperatures of the previous year and APIn for Elm, Cottonwood, and Mulberry, and early fall temperatures for Juniper. Linear regression models developed for Elm, Cottonwood, and Mulberry used the monthly mean maximum temperature for the month of June of the prior year as the independent variable to yield a *R* squared statistic (*R*^2^) of 0.88, 0.91 and 0.78, respectively. For Juniper, the average monthly mean minimum temperature for the previous September and October served as the independent variable and yielded the *R*^2^ value of 0.80. We also observed a positive trend for the annual maximum temperature over time and a negative trend for the total APIn. Summers in New Mexico are hot and dry, and they may be getting hotter and drier because of climate change. Our analysis predicts that climate change in this area may lead to reduced allergies if temperatures continue to increase and if precipitation patterns remain the same.

## 1 Introduction

The goal of this study is to identify correlations between allergenic pollen abundance and meteorological factors and to build prediction models for the annual pollen integrals (APIn) in Albuquerque, New Mexico. The annual pollen integrals are obtained by summing the average daily concentration over a whole year, day/m^3^ (Galán et al., 2017) in Albuquerque, New Mexico.

Several chronic conditions such as allergic rhinitis, allergic conjunctivitis and allergic asthma are associated with pollen allergies (Pawankar et al., 2011). Allergic rhinitis affects between 10% and 30% of the worldwide population (Pawankar et al., 2011) and approximately 30% of the USA population (Wheatley & Togias, 2015) causing significant health and financial impacts (Lo et al., 2019). Asthma is one of the most common chronic diseases in New Mexico. In a 2014 report supported by the Centers for Disease Control and Prevention (CDC), a USA governmental agency, it was reported that asthma prevalence among adults in New Mexico has steadily increased since the 2000s; by that year, approximately 9.6% of adults aged 18 and older in New Mexico had asthma and pollen was a common asthma trigger (Resnick, 2014). Current data from the same agency show that the number of asthma cases in adults over 18 peaked at 11.8% in 2016 and is in decline. In 2019 it was reported to be 8.4%.

The population of the state of New Mexico, USA, is affected by allergies yearlong; in particular, the location of this study, the City of Albuquerque, Bernalillo County, New Mexico, with its short cold winters and hot summers, large temperature fluctuations, clear skies and dry, windy weather was ranked in 2021 by the Asthma and Allergy Foundation of America (AAFA) as the seventy-fifth most allergic city in the USA overall and sixty-seventh for both fall and spring seasonal allergies (https://www.aafa.org/media/2933/aafa-2021-allergy-capitals-report.pdf/).

Pollen calendars are valuable tools for allergy sufferers and clinicians (Katotomichelakis et al., 2015). The allergenic potential of the most important pollen taxa in the Continental USA and Southern Canada region can be found in the pollen calendar and maps of allergenic pollen (Lo et al., 2019) based on data from 31 National Allergy Bureau (NAB) pollen stations in the continental USA and Canada from 2003 to 2017.

The City of Albuquerque Air Quality program (https://www.cabq.gov/airquality) and the New Mexico Public Health Tracking Program (a CDC program) provide a list of the most common pollen found in Bernalillo County (https://www.cabq.gov/airquality/todays-status/pollen/pollen-identification) and a list of vegetation in the area surrounding Albuquerque that is often associated with pollen allergies: Juniper (*Juniperus*), Elm (*Ulmus*), Ash (*Fraxinus*), Cottonwood (*Populus*), Mulberry (*Morus*), common weeds (*Chenopodiaceae*), Sage (*Salvia*), Grass (*Poaceae*) and Ragweed (*Ambrosia*).

There are many studies linking climate change with pollen release and thus with allergies. Zhang et al. (2015) found that the start of allergenic pollen seasons of representative trees, weeds, and grass in the USA has started 3 days earlier on average, and that the average peak and total annual daily count have both increased from 2001 to 2010 compared to the 1990s. Rising carbon dioxide (CO_2_) concentrations can also affect the amount of pollen in the atmosphere (Singer et al., 2005; Ziska & Caulfield, 2000; Ziska et al., 2003).

Ziska et al. (2019) used global datasets with 20 years or more of airborne pollen across 17 locations in three continents to find that rising global temperatures may be contributing to extend the season duration and pollen intensity for several aeroallergenic pollen taxa.

Anderegg et al. (2021) using long-term pollen data from 60 North American stations from 1990 to 2018 found widespread advances and lengthening of pollen seasons and increases in pollen concentrations. Similar results were observed in other studies (Stinson et al., 2016; Velasco-Jiménez et al., 2020; Ziska et al., 2011).

Most recently, Levetin (2021) examined airborne pollen, temperature and precipitation in Tulsa, Oklahoma, which is close to New Mexico, for the eight most abundant pollen types. The results showed a significant increase in annual maximum temperature for the studied period with a nonsignificant trend toward increasing total pollen and a significant increase in tree pollen over time. In regard to specific taxa, the study found a significant increase in spring *Cupressaceae* and *Quercus* pollen, while *Ambrosia* pollen showed a significant decrease. A similar positive trend for trees was observed in a study by Gehrig and Clot (2021).

Wallace and Wang (2021) explored the seasonalities of influenza-like illnesses (ILIs), including COVID, and its relationship with the antibodies that pollens antigenically trigger in humans. They demonstrated close connections among global-scale atmospheric circulations, IgE antibody enhancement through seasonal pollen inhalation, and respiratory virus patterns at any populated latitude with a focus on the US.

Climate change is affecting New Mexico as the state is getting hotter and drier, with earlier springs, hotter summers, less predictable winters, and changing precipitation patterns with more intense droughts and a greater proportion of precipitation falling as rain rather than snow (Garfin et al., 2014).

The year 2020 was the second warmest year on record and the fourth driest in the state of New Mexico; for the city of Albuquerque, it was the eighteenth warmest and the twenty-third driest.

Many studies link preseason and in-season weather (Dąbrowska-Zapart et al., 2018; D’Amato et al., 2007; Flonard et al., 2018; Galán et al., 1998; García-Mozo et al., 2010; González Minero, et al., 1998; Myszkowska, 2014; Ocaña-Peinado et al., 2012; Rojo et al., 2021) with pollen. Ritenberga et al. (2018), tested a methodology for predicting next-year seasonal pollen index for large regions for birch in Northern and Northeastern Europe. Kurganskiy et al. (2021) found that grass pollen in Northwestern Europe was linked to preseason meteorological variables, particularly temperature and precipitation.

## 2 Materials and methods

### 2.1 Study site and climate

Albuquerque has a population of approximately 564,559 (2020 National Census https://www.census.gov/quickfacts/albuquerquecitynewmexico) with a total area of around 500 km^2^, 1% of which is water. Albuquerque lies in the center of the ecoregion of the Albuquerque Basin settled in a broad valley that stretches about 48 km east–west, bounded for most of its length by the Sandia Mountains to the east, the lower Manzano Mountains to the southeast, and low lava escarpments to the south and west.

The city is divided in half by the Rio Grande River with its Bosque Forest. The river follows the course of the Rio Grande Rift, a north trending continental rift zone separating the Colorado Plateau in the west from the North American Craton on the east, extending from southern Colorado to El Paso, Texas and flowing toward the Gulf of Mexico. Important cities within the rift north of Albuquerque and part of the region named Northern New Mexico with similar pollen allergies are Santa Fe, Española, home of Northern New Mexico College, Los Alamos, and Taos. Between these urban areas, the land is mostly rural and mountainous with different vegetation at high altitudes.

Albuquerque is surrounded by shrub and mesa vegetation, New Mexico cottonwoods in the Bosque, with the dominant species being *Populus deltoides* spp. wislizenii and piñon and juniper in the mountainous areas. Specifically, adjacent to the city, the Colorado Plateau semidesert, New Mexico mountains are forested with one seeded juniper and Rocky-Mountain juniper (*Juniperus monosperma* and *Juniperus scopulorum*), piñon (*Pinus edulis*), desert live oak (*Quercus turbinella*), gray oak (*Quercus grisea*) among others. The number of cottonwoods in the Bosque declined in the past due to human intervention. Restoration of the ecosystem, including the replanting of cottonwoods, is underway.

Mulberry and juniper trees are important pollen producers in Albuquerque. *Juniperus* pollen is highly allergenic and is produced in large quantities not only in New Mexico but in Texas and Oklahoma (Bunderson & Levetin, 2014). *Juniperus monosperma* is one of the most allergenic species of *Cupressaceae* in North America (Rogers & Levetin, 1998) and is present in south central Colorado, much of New Mexico, Arizona, the panhandles of Oklahoma and of Texas as well as southwestern Texas (Adams, 2008).

Mulberry trees have been a popular species for landscaping in Albuquerque, but male mulberries produce a high amount of pollen in the spring. Sensitization to Mulberry pollen has been reported as both a food allergy and a respiratory allergy (Papia et al., 2020). In regard to cottonwoods, *Populus* pollen is considered a minor allergen; however, sufficient airborne pollens quantities can provoke sensitization in humans (Costache et al., 2021).

The city has abundant non-native plants such as Siberian elm, Russian olive, salt cedar, and mulberries. Albuquerque has outlawed the planting of certain trees to control their allergenic pollen, first in the 1994 Albuquerque Pollen Control Ordinance and in the Amendment to the Pollen Ordinance in 2004. The City of Albuquerque also has a webpage entitled “Restricted Trees for Pollen Control”. The list of restricted trees includes all trees in the Genus *Ulmus, Morus*, and *Populus* (with one of the exceptions being the *Populus deltoides* ssp. *wislizeni*, Rio Grande cottonwood) and all male trees in the Genus *Juniperus*.

The climate in Albuquerque is cold semiarid, mostly sunny and dry, with most of the rainfall occurring during the summer monsoon season (North American Monsoon System or NAMS) which peaks in the month of July. The annual averages for rain and snow, humidity and daily mean temperature are 240 mm, 241 mm, 44%, and 14 °C, respectively. Albuquerque is a windy city. The wind comes most often from the west from the end of September to the end of June, and from the south in the three months of summer.

**Fig. 1:**
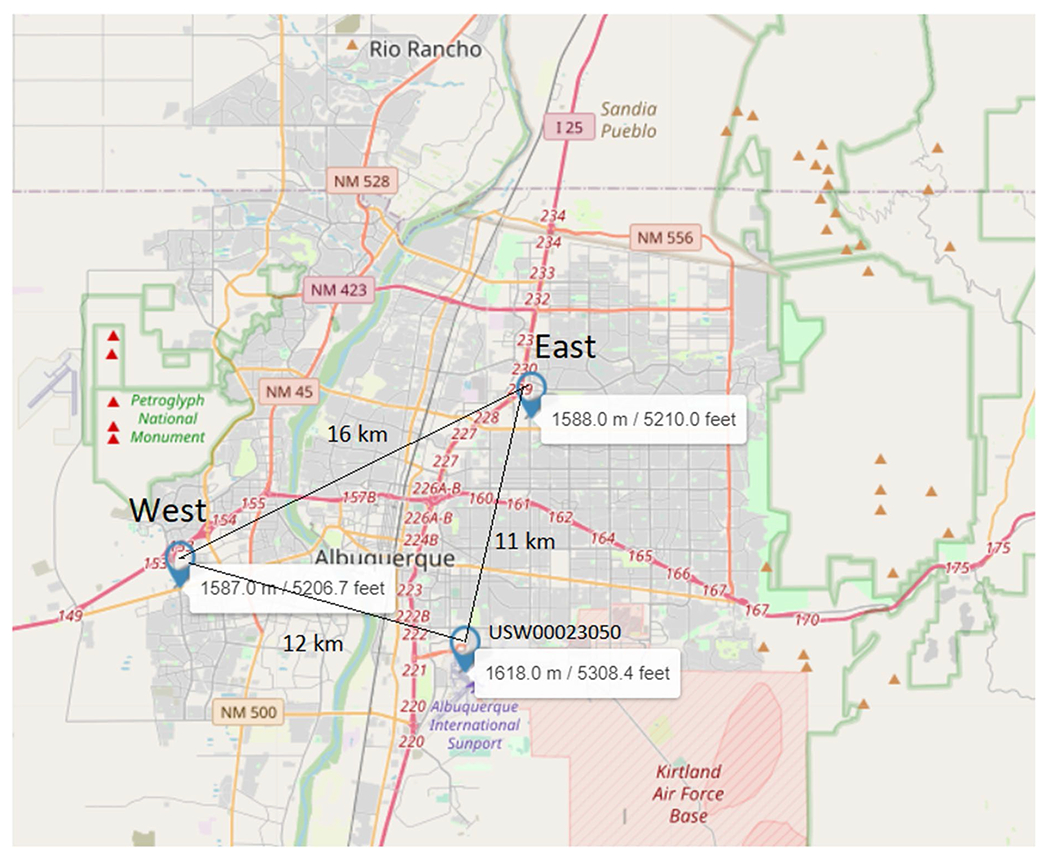
West and east samplers and weather station (at Albuquerque International Airport) USW00023050. The three points form an approximate isosceles triangle with the two samplers being almost equidistant from the weather station. The west sampler is exposed to the desert environment at the edge of the urban part of the city. The east sampler is closer to the Sandia Mountains that run north–south to the east of the sampler

The pollen data used in this study from 2004 to 2020 were collected by the City of Albuquerque using a Burkard Volumetric Spore Trap. The city manages two pollen samplers located within 16 km of each other: the east sampler at 1588 m, latitude/longitude 35.13426,– 106.58593 and the west sampler at 1587.0 m, latitude/longitude 35.07267,– 106.74275 (Fig. 1).

The city chose these locations so that the east sampler samples from a more urban environment while the west sampler samples from a typical desert environment. The east sampler data set is incomplete and does not include the consecutive years from 2012 through 2015. In this study, we used data from the west sampler since it is more representative of the Northern New Mexico environment. In addition, Juniper, one of the most prevalent sources of allergies during the end of winter and spring in Northern New Mexico and not just Albuquerque, is ubiquitous in the natural environment, and the west sampler we presume will map this type of pollen better. The east sampler was used for comparison purposes.

Data from these two stations differ for some years. Since the west station is closer to the open land and since some of the trees are specifically planted within the confines of the urban area (with the exception of trees like juniper, cottonwoods, and types of oak) we believe the difference between the two stations is due primarily to the wind conditions. If the wind comes from the west, south or northwest, the west station may receive little of the airborne pollen coming from the city. The role of the wind in addition to cold temperatures has been mentioned in several reports from the city to explain why counts from the west sampler in certain years are much lower than counts in the east sampler. We can also speculate that city pollen may be trapped longer than usual within the city due to the barrier the Sandia Mountains to the east present when winds blow toward the mountain. Wallace and Wang (2021), using data from both samplers, reached similar conclusions when noticing a lower pollen count in the west sampler. According to Flonard et al. (2018), the variability of pollen data for *Cupressaceae* in Tulsa, Oklahoma, depends on the strength and direction of winds. Pollen grains can be transported hundreds of kilometers in the atmosphere (Rogers & Levetin, 1998; Van de Water & Levetin, 2001), but local pollen is still the most important contributor (Keynan et al., 1991; Lo et al., 2019; Ranta et al., 2006).

### 2.2 Meteorological data

The meteorological data were collected from the Albuquerque International Airport weather station, which is part of a network of stations for the National Oceanic and Atmospheric Administration (NOAA) Climate Data Online portal (https://www.ncdc.noaa.gov/cdo-web/). This station is at the southeast part of the city of Albuquerque, and it is located south of both pollen stations forming an approximate isosceles triangle (Fig. 1) whose sides are 12 km from the west sampler and 11 km from the east, Network ID GHCND: USW00023050, altitude 1618.5 m, latitude/longitude: 35.0419,– 106.6155.

The dataset used (collected from the NOAA station depicted in Fig. 1) is the Global Summary of the Month from the NOAA online portal, and the variables used in this study (see Table 1) are described in the NOAA data documents.

We extracted the monthly averaged meteorological variables *T*_MAX_, *T*_MIN_, *T*_AVG_, PRCP and AWND from the NOAA dataset for the period 2003–2020 to study correlations with APIn data for the period 2004–2020.

### 2.3 Pollen data

Albuquerque’s pollen season lasts from March 1 through October 1. This is based on information from the City of Albuquerque Air Quality Bureau. The data comes in the form of a time series that was transformed into the annual pollen integral (APIn). Common pollination periods by group are trees (late winter and spring), weeds (late spring and summer), and grass (summer and autumn).

The City of Albuquerque’s Air Quality program lists Juniper/Cedar, Elm, Ash, Cottonwood, Mulberry, Chenopodiaceae, Sage, Grass, and Ragweed as the vegetation most commonly associated with allergies. The City of Albuquerque also tracks other types of pollen, but we chose to focus on these types since they are the pollen types associated with allergies. In addition, after evaluating the data for the eight most allergenic genera described by the city, we found evidence of strong correlation with our proposed climate variables only for tree pollen. Therefore, we chose to perform a correlation analysis with the primary pollen producing trees in Albuquerque that cause allergies: *Morus* with pollen produced from April through May; *Juniperus* from January through April and September through December; *Ulmus* from January through April; and *Populus* both from March through June. We extracted pollen time series for the selected genera for the 2004–2020 period.

### 2.4 Methodology

With this data, we explored the effects of monthly averaged meteorological variables conditions prior to and within a pollen season on the APIn, or in other words, on the capacity of a plant or tree to create a reserve of pollen. To fully study the effects of meteorological conditions on a pollen season, we believe we needed to consider the meteorological variables from the previous year.

We tested the APIn for linear correlations with monthly averaged meteorological variables for the previous year and in the current year. Each of the meteorological variables was arranged into sets of months and tested against the APIn for 2004–2020. The tests were performed first with 1 year delay, from 2003 to 2019, starting from the month of May in 2003 and then, using the current year from 2004 to 2020 beginning on January up to the end of the season.

We also considered the average of two consecutive months as an independent variable. This was done since abrupt changes in temperature and precipitation are very common in New Mexico and may occur in transitions between two months. In all, considering the months (April of the previous year to August of the current current) and bimonthly averages (April and May of the previous year to February and March of the current year), there were 29 time periods. Since there were five meteorological variables, we created a total of 5 × 29 = 145 independent variables for testing (by forming all combinations of months and monthly averages with each of the five meteorological variables).

A MATLAB code was written to test each of the variables against APIn using Regress, a multiple linear regression tool that calculates the least squares line and provides statistical information. Regress returns 95% confidence intervals for the model coefficients and 5% significance levels for the residual intervals. From the pool of data, we looked for outstanding cases with high values of *R*^2^. Some years were excluded from the analysis either through (a) visual inspection if the year was very irregular (for example the pollen for *Ulmus* and *Populus* was unusually high in 2020 and dwarfed the APIn from previous years), or (b) by being flagged as an outlier by the MATLAB code. We used the method described in Regress to diagnose outliers which uses residual intervals that do not contain zero. The complete list of outlying year(s) that were excluded for trees were 2011, 2014 and 2018 for *Juniperus*, 2020 for *Populus* and *Ulmus*, and 2005 and 2020 for *Morus*.

## 3 Results and discussion

### 3.1 Analysis of the input data

**Fig. 2:**
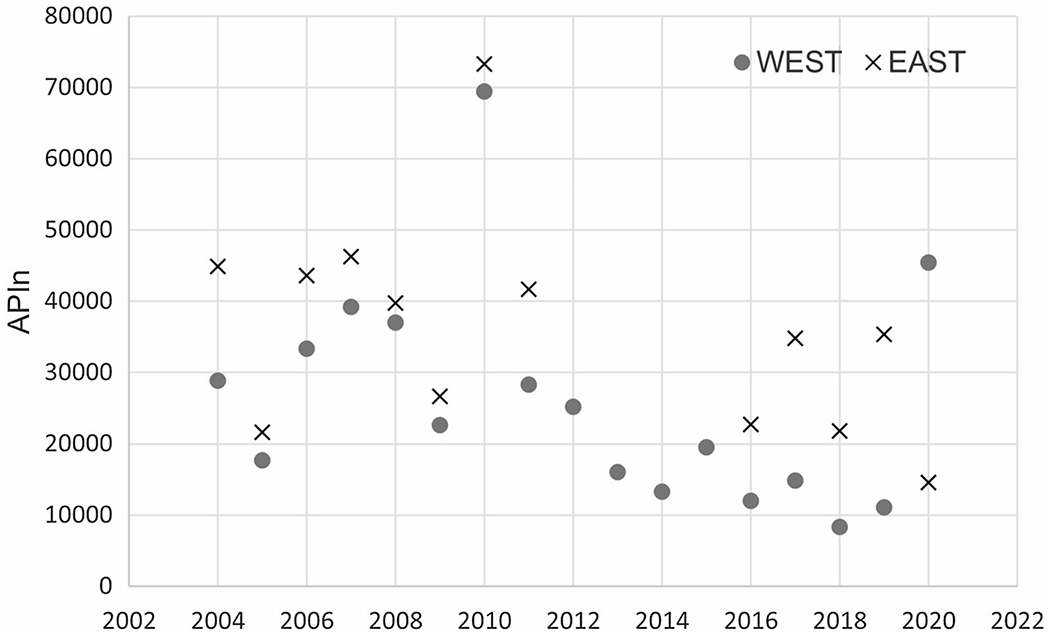
Time series of APIn from west and east samplers for the years 2001 to 2020. The east sampler is missing years from 2012 to 2015

Table 2 shows the total APIn for each year for the west and east samplers for selected taxa along with meteorological variables *T*_MAX_, *T*_MIN_ and PRCP. Table 3 shows the percentage contribution for the selected pollen type for the period 2014–2020. Our first observation is that the total APIn seems to decrease with time in the west sampler except for years 2010 and 2020. The decrease is also observable the east sampler (even though some years are missing). See Fig. 2. This trend was also observed by Wallace and Wang (2021). We also note that the mean annual average of *T*_MIN_ exhibits a positive annual trend over time with a linear regression slope of 0.08 and a *R*^2^ value of 0.39. The maximum temperature *T*_MAX_ in Albuquerque also shows a positive upward trend over time. Thus, the total APIn seems to decrease as *T*_MIN_ and *T*_MAX_ increase in Albuquerque. This is unusual since many other studies (e.g., Levetin, 2021) observe an increase in pollen in the Southeast USA where maximum temperatures have been increasing.

While Table 2 shows that the total APIn changed significantly during the study period, Table 3 column 2 shows that *Cupressaceae (27%), Morus (22%)*, and *Chenopodiaceae (17%)* contribute the most pollen for most of the 17 recent years followed by *Salvia* (9%) and *Poaceae* (7%). Not all taxa are shown in the table. Although fluctuations are observed from year-to-year for all pollen types, *Morus* has changed drastically. *Morus* contributed 37% to the total pollen from 2004 to 2010 (column 3) but only 12% from 2011 to 2020 (column 4).

Comparatively, the ranking of percentage contribution to total pollen for the Continental USA and Southern Canada Pollen Calendar in Lo et al. (2019) begins with *Quercus* (genus) at 19.6%, *Cupressaceae* (family) 19.4%, *Ambrosia* (genus) 7.2%, and *Morus* (genus) 6.7% as the first four. New Mexico was not included in the 31 stations used in that calendar.

**Fig. 3:**
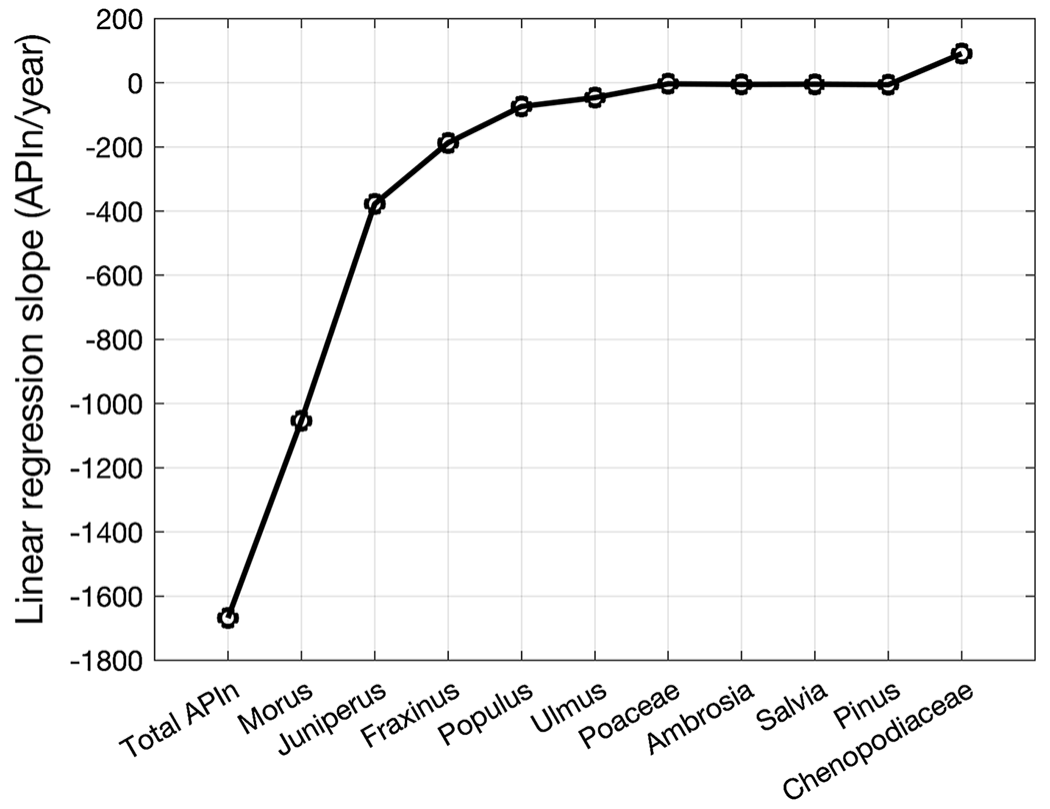
Linear regression slope constructed from the annual pollen index (APIn) for the years 2004 to 2019

Figure 3 shows the linear regression slope constructed from the annual pollen index (APIn) for the years 2004 to 2019. The slope of the linear regression line is negative for the total pollen APIn versus *Morus, Juniperus, Fraxinus, Populus*, and *Ulmus*, slightly negative for *Poaceae, Ambrosia, Salvia*, and *Pinus*, and positive for common weeds (*Chenopodiaceae*). The total pollen APIn sums the APIn values for all pollen types shown in the figure.

### 3.2 Modeling results

**Fig. 4:**
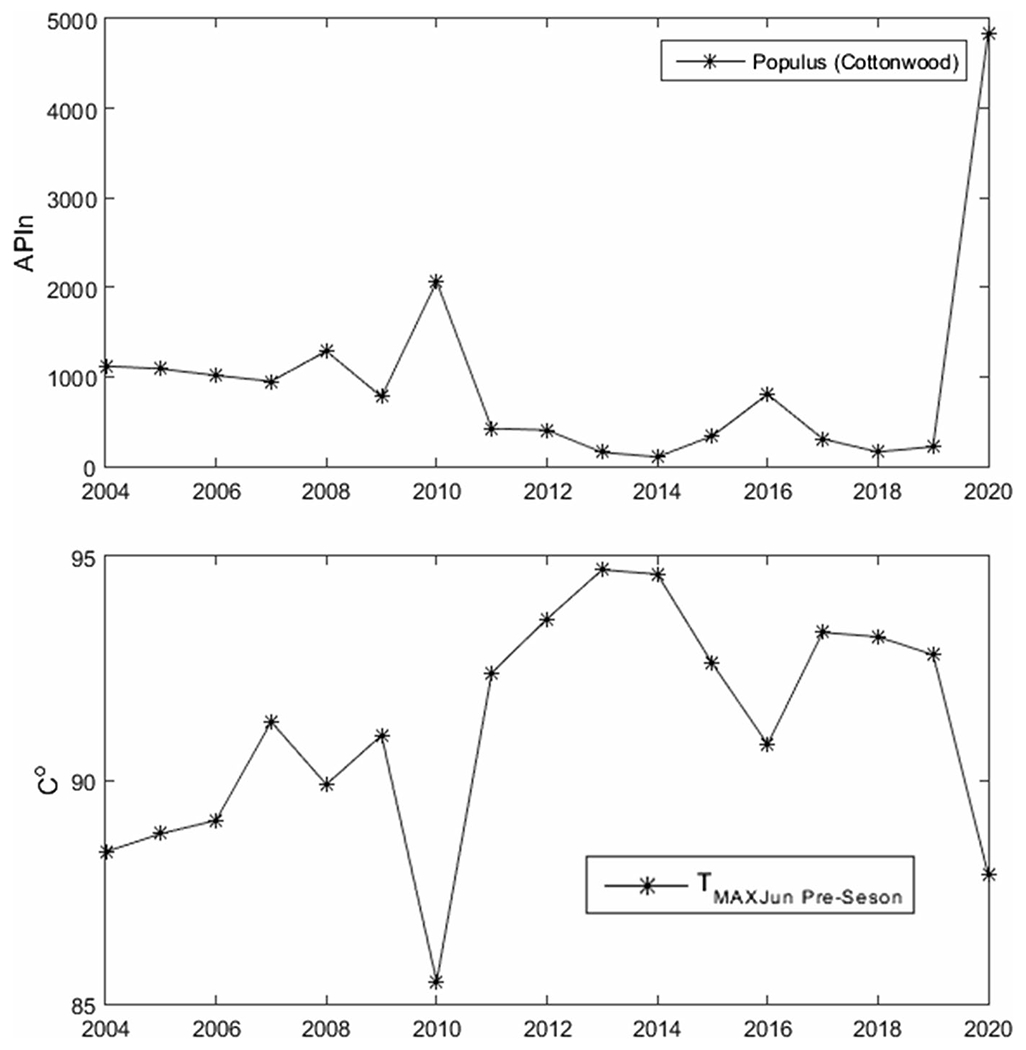
APIn and *T*_MAX_ from June of the summer prior for Populus for the years 2004 to 2020

**Fig. 5:**
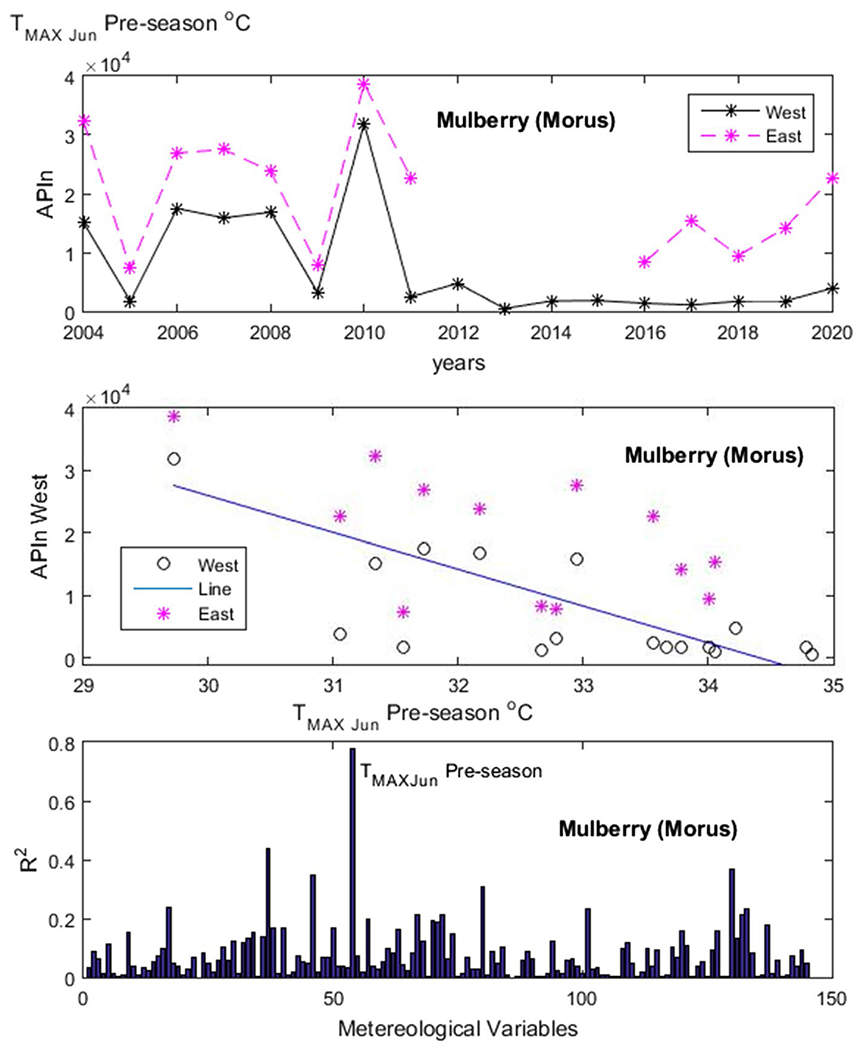
Morus. Panels from top to bottom: time series of APIn for the west and east samplers for comparison purposes; linear model derived from the west sampler (line) and data from both samplers; and *R*^2^ results for all variables run by our code. The outstanding cases are noted. See detailed description in text

**Fig. 6:**
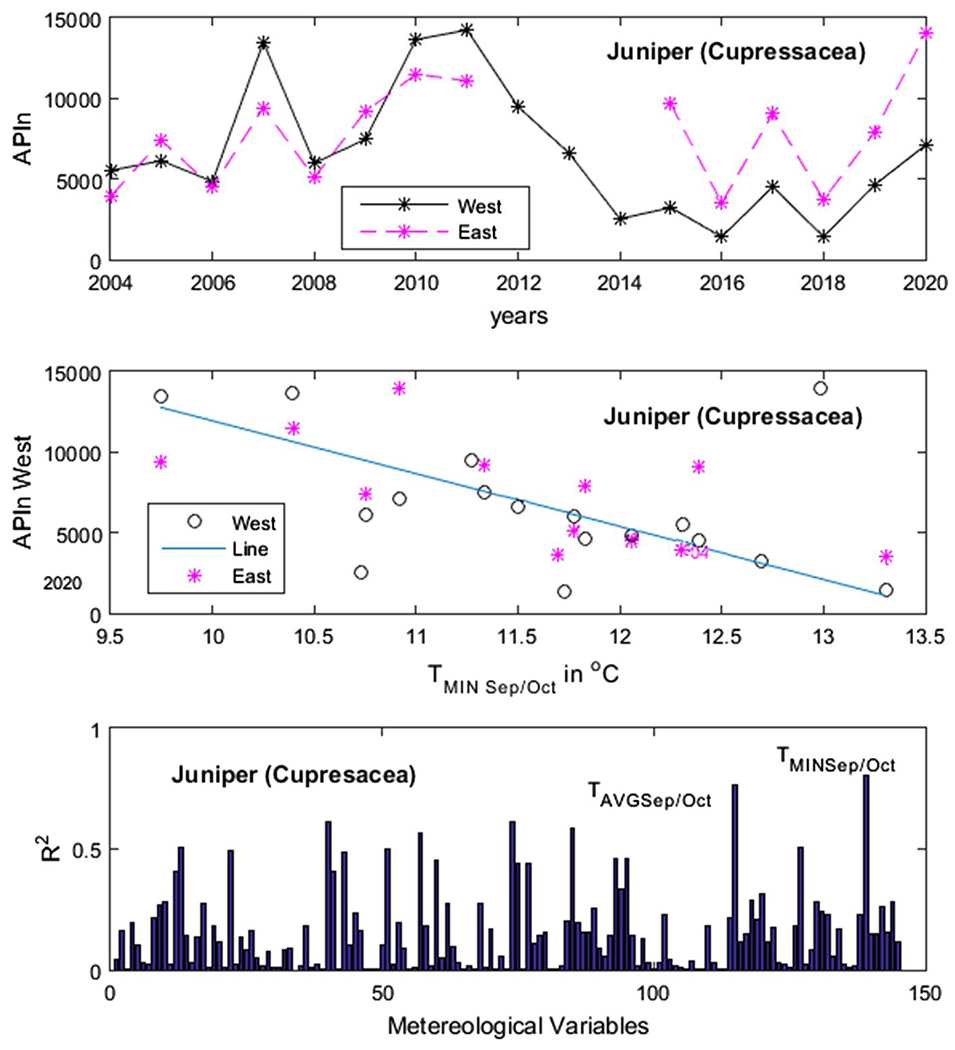
Juniperus. Panels from top to bottom: time series of APIn for the west and east samplers for comparison purposes; linear model derived from the west sampler (line) and data from both samplers; and *R*^2^ results for all variables run by our code. The outstanding cases are noted. See detailed description in text

**Fig. 7:**
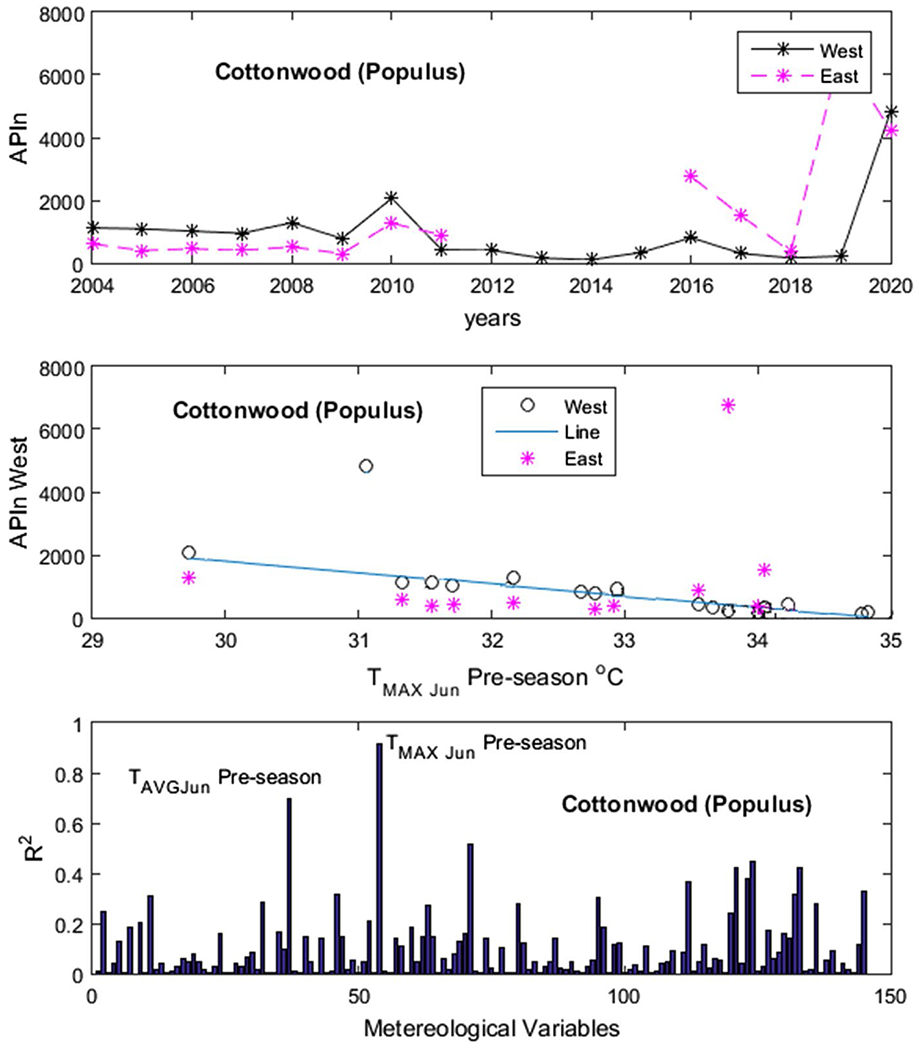
Populus. Panels from top to bottom: time series of APIn for the west and east samplers for comparison purposes; linear model derived from the west sampler (line) and data from both samplers; and *R*^2^ results for all variables run by our code. The outstanding cases are noted. See detailed description in text

**Fig. 8:**
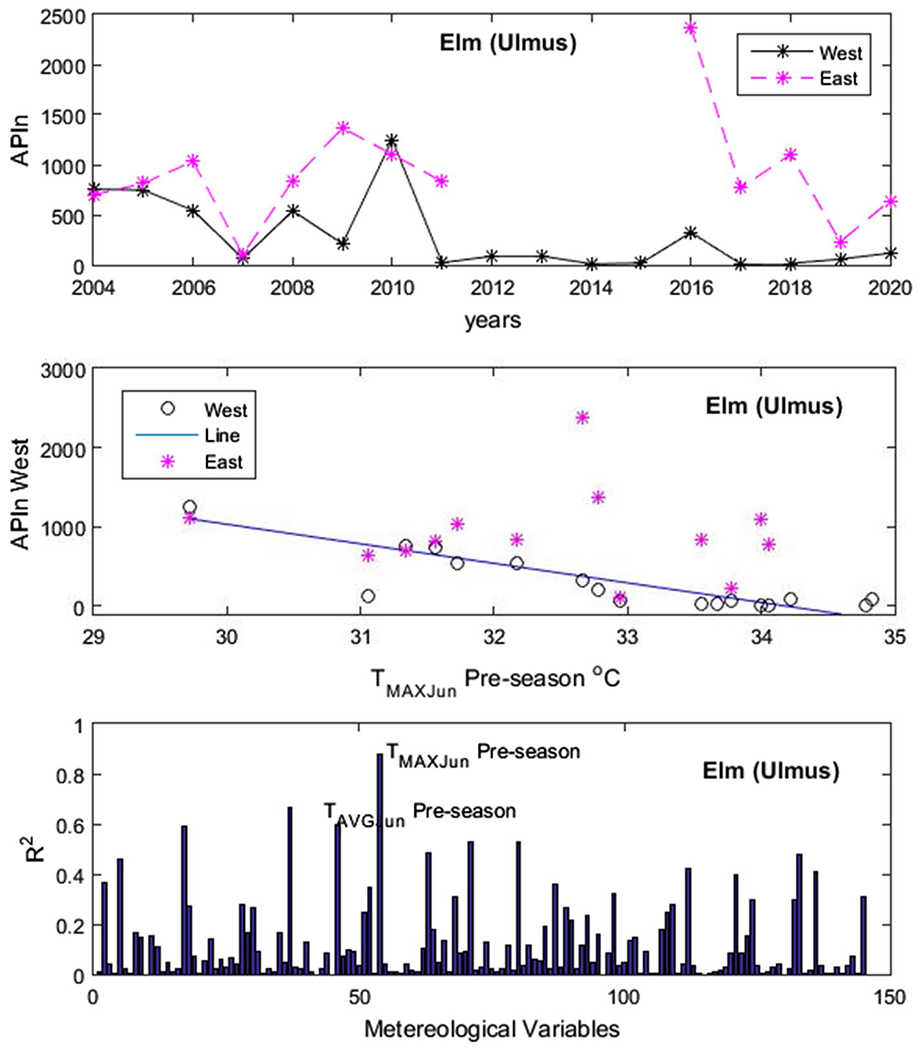
Ulmus. Panels from top to bottom: time series of APIn for the west and east samplers for comparison purposes; linear model derived from the west sampler (line) and data from both samplers; and *R*^2^ results for all variables run by our code. The outstanding cases are noted. See detailed description in text

Figure 4 shows an example of our results for *Populus*. In the upper panel, we have the APIn for the years 2004 to 2019 and in the lower panel one of the variables used for testing—the monthly average maximum temperature *T*_MAX_ for the month of June of the previous year, which would be the beginning of the summer prior. The variable *T*_MAX_ in June of the previous year was the one with the highest *R*^2^. In Figs. 5, 6, 7 and 8, the first panel shows, for comparison, values of APIn for the west station (solid lines) and for the east station that was not used (dashed line and magenta color). The second panel shows the resulting linear model, the west sampler APIn values in black (circles for the data that was used) and the east values in magenta. The third panel shows the resulting *R*^2^ for all variables for each genus. Only the outstanding cases are labeled.

We note that strong and moderate La Niña events occurred in the periods 2007–2008, 2010–2011 and 2020–2021; meanwhile 2014 and 2018 were exceptionally neutral years with no El Niño or La Niña events. We will investigate possible links in future work.

For all the trees in this study, except for *Fraxinus*, we found one or two outstanding meteorological variables (> 0.70) with good linear correlations. If two variables were present, *T*_AVG_ was one of the variables and *T*_MIN_ or *T*_MAX_ was the second. None of the variables reached an *R*^2^ value greater than 0.62 for *Fraxinus*. We also observed a sharp decline in APIn for *Fraxinus* after 2010 but only in the west sampler. Since we are not able to explain such huge discrepancies in Ash pollen, we chose not to include *Fraxinus* in Table 4 results.

We conclude that a linear trend was present for *Juniperus, Ulmus, Populus* and *Morus*. The results are shown in Table 4. We did not run the linear model analysis for the east sampler due to a gap in the data, but we did test the east sampler data for a linear correlation using the best variable from the west sampler. We note that the Pearson *R* values for the west and east sampler agree in sign for all genera except for *Populus*. Again, we do note that other sources (e.g., Ziska et al., 2019) found that annual cumulative increases in *T*_MIN_ were positively associated with increases in seasonal pollen load (*R* = 0.61, *p* = 0.010) in locations across the northern hemisphere. Thus, since the amount of pollen in Albuquerque decreases with temperature, Albuquerque does present a unique environment for pollen production.

In Fig. 5 *Morus*: The most important variable was *T*_MAX_ in June of the previous year with *R*^2^ = 0.78. In Fig. 6 *Juniperus*: There were two variables with *R*^2^ values above 0.70: *T*_MIN_ for the bimonthly average of September and October of the previous year (*R*^2^ = 0.80) and *T*_AVG_ for the same months (*R*^2^ = 0.76). In Fig. 7 *Populus*: There were two variables with significantly higher *R*^2^ values: *T*_MAX_ (*R*^2^ = 0.91) and *T*_AVG_ (*R*^2^ = 0.70) for the month of June of the previous year. The year 2020 was excluded. Both stations show similar APIn. Cottonwoods (*Populus*) are present within and outside the city. In Fig. 8 *Ulmus*: *R*^2^ = 0.88 with *T*_MAX_ in June of the previous year and a lesser one *T*_AVG_ (*R*^2^ = 0.66) of the same months.

For *Juniperus* and *Morus*, the relative error of the linear regression constants was high in both cases (Table 4, column 3). We need to mention that although we decided not to include *Fraxinus* in our correlation results, the highest *R*^2^ of 0.62 for *Fraxinus* corresponded to *T*_MAX_ for June of the year prior which was the meteorological variable with the highest *R*^2^ for *Morus, Ulmus*, and *Populus*.

**Fig. 9:**
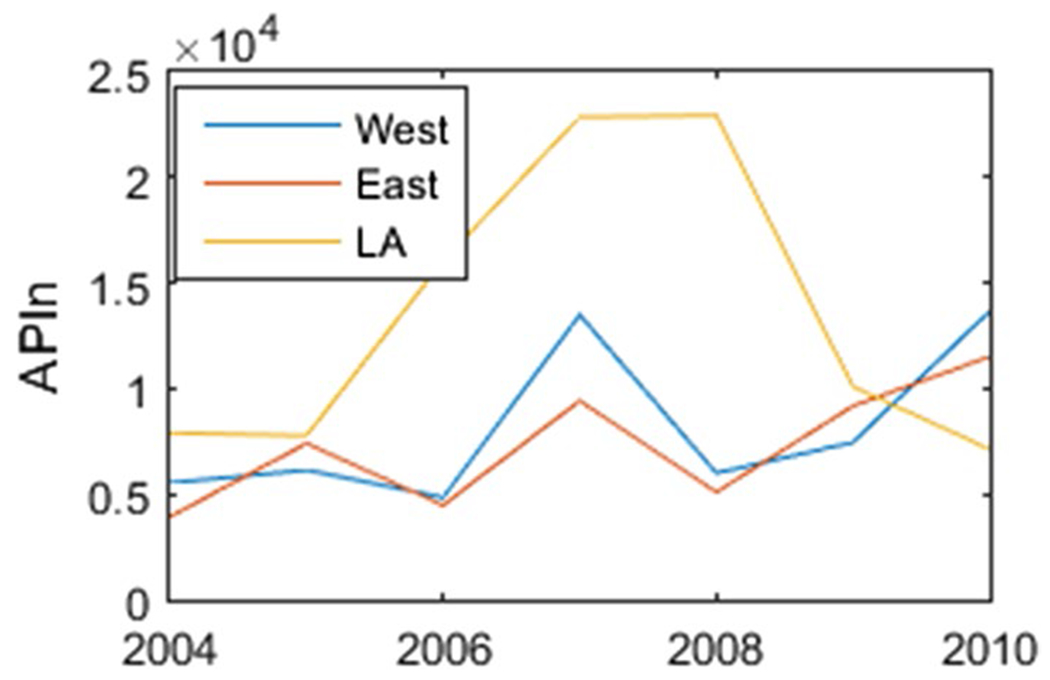
Time series of APIn for Cupressaceae: Comparison between the two samples for Albuquerque, west and east, and pollen data from the city of Los Alamos, 93 km north of Albuquerque, located in the Jemez Mountains 660 m higher than Albuquerque

Since *Juniperus* is ubiquitous to New Mexico and not just confined to the city limits, we wanted to explore if changes we see in the two samplers, west and east, for Albuquerque can be also observed in a city of Los Alamos (latitude/longitude: 35.8800, – 106.3031), 94 km north of Albuquerque and located in the Jemez Mountains at an elevation of 2231 m for 2004 to 2010. *Juniperus* is more prevalent in this area, when compared to the data from the city. Since similar behavior is observed in Fig. 9, we can infer that the response to any change from *Cupressaceae* is also related to the Northern New Mexico area rather to the city itself.

In all the four cases noted in Table 4, the correlation was negative for the west sampler. Among all possible independent variable combinations, *T*_MAX_ for the month of June (from the previous summer) showed the highest linear correlation for *Ulmus, Populus*, and *Morus*. For *Juniperus*, the average of *T*_MIN_ for September and October generated the highest *R*^2^ but other combinations (different meteorological variable at different times) with lesser *R*^2^ were present (Fig. 6) and may be considered for a multivariable linear model in future studies.

Our forecasting model for *Juniperus* seems to predict, with some outliers, that cooler temperatures at the start of the fall increase pollen production. Preseason meteorological variables have been proven to be important for pollen maturation and release in many spring-pollinating tree species as warmer winters were linked to earlier pollen seasons. The extensive study of Juniper in Tulsa, Oklahoma, which is close to New Mexico by Levetin (2021), mentions that more work needs to be devoted to investigating the influence of preseason meteorology on *Cupressaceae* to help build forecasting models. Cariñanos et al. (2004), calculated Spearman’s correlation between the daily concentrations of the eight most representative pollen taxa in the atmosphere above Chirivel, Spain, and daily values of average temperature and precipitation during the 6-year period of study. They found a strong negative correlation with temperature for *Cupressaceae*. They attributed this to the occurrence of frosts during the flowering periods resulting in conditions suitable for airborne pollen presence in the atmosphere.

Earlier hot summers seem to work against the production of pollen for the next season for *Ulmus, Populus*, and *Morus* trees. We do not have an explanation for this occurrence, but we can argue that since the most highly correlated meteorological variable occurs many months before the flowering season, the development of the plant is probably affected. For these trees, we would also like to explore a multivariable model in the future that includes the occurrence and timing of precipitation. Other factors affecting counts in the samplers which cannot be dismissed include the wind and the city bans on trees.

## 4 Conclusion

Our study has shown that the maximum temperature for the month of June (*T*_MAX_) for Cottonwood (*Populus*), Elm (*Ulmus*), and Mulberry (*Morus*), and the bimonthly average for minimum temperature for the months of September and October ((*T*_MIN_(Sept) + *T*_MIN_(Oct))/2) for Juniper (*Juniperus*) have a negative correlation with the annual pollen counts APIn for the subsequent season. Meteorological conditions of the current season show comparatively weaker correlations with APIn. These results are unique to Albuquerque, New Mexico, since many studies show a positive correlation with pollen and temperature (Levetin, 2021; Ziska et al., 2019).

We conclude that the monthly average maximum temperature 6 months prior to the onset of the season is correlated with the APIn released for *Ulmus*, and 8–9 months prior to the season for *Populus* and *Morus*. The monthly average of the minimum temperature for September and October 6 months prior to the season was correlated with the APIn for the first *Juniperus* season. While our correlation and linear regression study identified the monthly meteorological variable(s) with the highest *R*^2^ value, we believe that it could benefit from a multiple regression analysis. Although 2020 was an unusual year, our model predicts that the pollen counts for all four tree genera may continue to decrease.

The negative correlation between temperature and total of all APIn taxa also suggests that fewer allergies will occur since pollen is an allergy trigger. The percent of adults with allergies in Albuquerque has decreased recently.

We do acknowledge that the impact of our model predictions may be affected by recent climate changes. For example, 2020 and 2021 were labeled La Niña years. Climate change will continue to change what is considered normal for New Mexico by impacting pollen and allergy seasons. We hope that we have helped identify important meteorological variables that influence pollen production in Albuquerque, New Mexico.

## Figures and Tables

**Table 1:** Definition of meteorological variables

*T*_MAX_: Monthly mean maximum temperature – Average of daily maximum temperature
*T*_MIN_: Monthly mean minimum temperature – Average of daily minimum temperature
*T*_AVG_: Average monthly temperature – computed by adding the unrounded monthly mean *T*_MAX_ and *T*_MIN_ and dividing by 2
PRCP: Total monthly precipitation. Precipitation totals are based on daily or multi-day (if daily reports are missing)
AWND: Monthly average wind speed based on the daily AWND values from the Global Historical Climatology Network (GHCN-D)

**Table 2:** Total annual pollen integral (APIn) per year for the west and east stations and the three variables used in the study: *T*_MAX_ for monthly mean maximum temperature, *T*_MIN_ for monthly mean minimum temperature, and PRCP for total monthly precipitation

Year	APIn total west	APIn total east	*T*_MAX_ (Celsius)	*T*_MIN_ (Celsius)	PRCP (mm)
2004	28,832	44,916	20.4	7.6	300
2005	17,645	21,593	21.2	8.3	290
2006	33,303	43,604	21.1	7.8	332
2007	39,186	46,246	21.2	7.7	260
2008	36,975	39,765	20.9	7.2	212
2009	22,596	26,669	21.3	7.6	170
2010	69,438	73,309	21.3	7.8	228
2011	28,328	41,707	21.7	7.4	120
2012	25,142	N/A	22.7	8.3	139
2013	16,055	N/A	21.1	7.3	237
2014	13,309	N/A	21.8	7.8	224
2015	19,541	N/A	21.5	8.1	292
2016	11,972	22,753	22.1	8.1	170
2017	14,831	34,846	22.7	8.6	195
2018	8285	21,840	22.1	8.1	222
2019	11,085	35,369	20.9	7.3	223
2020	45,440	14,562	22.3	7.8	149

The N/A refers to missing data for the east sampler (2012 to 2015)

**Table 3:** Average percentage contribution for some selected taxa: Column 2 from 2004 to 2020, Column 3 from 2004 to 2010, and Column 4 from 2011 to 2020

Taxa (selected)	2004–2020 (%)	2004–2010 (%)	2011–2020 (%)
*Juniperus, Cupressaceae*	27	25	26
*Morus*	22	37	12
*Chenopodiaceae*	17	8	23
*Salvia*	9	6	12
*Poaceae*	7	4	10
*Fraxinus*	5	7	3
*Pinus*	4	2	4
*Populus*	3	4	3
*Ambrosia*	2	1	3
*Ulmus*	1	2	1
*Quercus*	1	0	1
*Other/unidentified*	2	4	2

Different time periods are displayed to show fluctuations over time. Taxa that were deemed of little individual contribution or missing for some years and unidentified pollen were grouped together as “Other/Unidentified”

**Table 4:** Linear regression analysis of tree pollen

Pollen type	Variable with outstanding *R*^2^ for West sampler	West sampler (2004–2020) linear model: *y*(*x*) = APIn *y* = *b*0 + *b*1 *x* and Pearson correlation coefficient *R*	East sampler Pearson correlation coefficient with the variable listed in the second column
*Morus*	*x* = *T*_MAX_ Jun Excluded: 2005 and 2020	*b*0 = 202555 ± 30% *b*1 = −5886 ± 38% *R*^2^ = 0.78 *p* = 1.4 × 10^−5^ *R* = −0.8813	*R* = −0.8181 Excluded: 2005 and 2020
*Juniperus*	*x* = *T*_MIN_ average Sep and Oct excluded: 2011, 2014, and 2018	*b*0 = 44767, ±26% *b*1 = −3283 ± 31% *R*^2^ = 0.80 *p* = 1.3 × 10^−5^ *R* = −0.8974	*R* = −0.5785 Excluded: 2011, 2014, and 2018
*Populus*	*x* = *T*_MAX_ Jun Excluded: 2020	*b*0 = 12774 ± 16% *b*1 = −366 ± 17% *R*^2^ = 0.91 *p* = 7 × 10^−8^ *R* = −0.9566	*R* = 0.2933 Excluded: 2020
*Ulmus*	*x* = *T*_MAX_ Jun Excluded: 2020	*b*0 = 8423 ± 21% *b*1 = −246 ± 21% *R*^2^ = 0.88 *p* = 9 × 10^−8^ *R* = −0.9373	*R* = −0.2731 Excluded: 2020
